# Monte Carlo investigation of collapsed versus rotated IMRT plan verification

**DOI:** 10.1120/jacmp.v15i3.4681

**Published:** 2014-05-08

**Authors:** Elaine Conneely, Andrew Alexander, Russell Ruo, Eunah Chung, Jan Seuntjens, Mark J. Foley

**Affiliations:** ^1^ School of Physics National University of Ireland Galway Galway Ireland; ^2^ Medical Physics Unit McGill University, Montreal General Hospital Montreal QC Canada

**Keywords:** Monte Carlo, IMRT, quality assurance

## Abstract

IMRT QA requires, among other tests, a time‐consuming process of measuring the absorbed dose, at least to a point, in a high‐dose, low‐dose‐gradient region. Some clinics use a technique of measuring this dose with all beams delivered at a single gantry angle (collapsed delivery), as opposed to the beams delivered at the planned gantry angle (rotated delivery). We examined, established, and optimized Monte Carlo simulations of the dosimetry for IMRT verification of treatment plans for these two different delivery modes (collapsed versus rotated). The results of the simulations were compared to the treatment planning system dose calculations for the two delivery modes, as well as to measurements taken. This was done in order to investigate the validity of the use of a collapsed delivery technique for IMRT QA. The BEAMnrc, DOSXYZnrc, and egs_chamber codes were utilized for the Monte Carlo simulations along with the MMCTP system. A number of different plan complexity metrics were also used in the analysis of the dose distributions in a bid to qualify why verification in a collapsed delivery may or may not be optimal for IMRT QA. Following the Alfonso et al.^(1)^ formalism, the $k_{Q_{clin},Q}^{f_{clin},f_{ref}}$ correction factor was calculated to correct the deviation of small fields from the reference conditions used for beam calibration. We report on the results obtained for a cohort of 20 patients. The plan complexity was investigated for each plan using the complexity metrics of homogeneity index, conformity index, modulation complexity score, and the fraction of beams from a particular plan that intersect the chamber when performing the QA. Rotated QA gives more consistent results than the collapsed QA technique. The $k_{Q_{clin},Q}^{f_{clin},f_{ref}}$ factor deviates less from 1 for rotated QA than for collapsed QA. If the homogeneity index is less than 0.05 then the $k_{Q_{clin},Q}^{f_{clin},f_{ref}}$ factor does not deviate from unity by more than 1%. A value this low for the homogeneity index can only be obtained with the rotated QA technique.

PACS number: 87.55.Qr

## I. INTRODUCTION

In order for intensity‐modulated radiotherapy (IMRT) treatments to achieve their potential, it is necessary to be able to accurately verify the radiation dose that will be administered to the patient in these treatment techniques. Previous studies have shown that standard treatment planning methods in certain clinical IMRT configurations are limited in the prediction of the radiation dose. For example, Ibbott et al.^(2)^ reported that roughly 30% of institutions failed to deliver a dose distribution to a head and neck phantom that matched their own treatment planning system dose to within 7% for dose or 4 mm distance to agreement.

There are many reports documenting the limitations of commercial treatment planning algorithms in planning IMRT treatments, especially in complicated situations such as for head and neck cancers.^3^, ^4^ It has been determined that as many as 46% of patients receive a maximum dose that is more than 10% higher than the prescribed dose, and 63% of patients receive a dose that is more than 10% lower than the prescribed dose.^(4)^ This finding has very substantial implications for the treatment of cancer patients using IMRT, as a dose 5% lower than the prescribed dose may result in clinically detectable reduction in tumor control. The International Commission on Radiation Units and Measurements recommends that radiation dose be delivered to within 5% of the prescribed dose.^(5)^

One of the primary issues with a more widespread implementation of intensity‐modulated radiation therapy (IMRT) in clinics with the necessary equipment is that it can be substantially more time‐consuming than conventional radiotherapy. Work by Miles et al.^(6)^ showed an average increase in physics man‐hours of 4.9 hours per patient treated. IMRT plan QA forms a vital part of the IMRT treatment process. Each individual IMRT patient plan is verified through a two‐step process, as advised by the AAPM,^(7)^ of dose verification of the plan on a homogeneous phantom:

1) measurement of the absorbed dose to a point in a high‐dose, low‐dose‐gradient region in a clinically substantial volume of the phantom with an ion chamber; and
2) the measurement of the two‐dimensional relative dose distribution, with either film, a diode array device, or an ion chamber array device, and a gamma analysis^8^, ^9^ of the resulting dose distribution when compared to the plan.

As this procedure is required for each IMRT patient plan before treatment commences, it can be seen how this would contribute to a substantial increase in time and personnel requirements. The measurement of the relative dose distributions is discussed in more detail in the work by Nelms et al.^(9)^ This work focuses on the measurement of the absorbed dose.

A recent survey by Nelms and Simon^(10)^ showed that a noteworthy proportion of clinics (32.8%) use the single‐gantry‐angle composite (SGAC) technique for verification of 75%‐100% of patient IMRT plans. With such a comparatively high number of clinics using this single‐gantry‐angle practice, it would seem to warrant further investigation and verification as an IMRT QA technique.

Alfonso et al.^(1)^ introduced a new approach for non‐standard beam reference dosimetry. For the calculation of dose for composite fields they recommend the use of an intermediate calibration field which they call a plan‐class‐specific reference field (PCSR). This is closer to the patient‐specific clinical fields and should provide a uniform dose over a region exceeding the dimensions of the reference detector. A correction factor is provided from this to compensate for differences between the standard calibration field and the small, composite fields used in IMRT. This is done to try to compensate for the deviations from charged particle equilibrium that can have an effect when using small fields, as is particularly applicable for IMRT QA. This is not accounted for in conventional dosimetry protocols which are based on the absorbed dose‐to‐water calibration at a reference field, usually a $10\times 10\;{\text{cm}}^{2}$ field.

This work aims to use Monte Carlo methods to investigate the reliability of using a single gantry angle or collapsed beam configuration, as opposed to measuring the doses at the planned angles or rotated beam delivery, when measuring the absorbed dose to a point in a high‐dose, low‐dose‐gradient region.^(11)^ The work in this paper aims to quantify the $k_{Q_{clin},Q}^{f_{clin},f_{ref}}$ factor for collapsed and rotated beam deliveries. The investigation looks at different plan complexity metrics to determine a predictor of the reliability of the QA technique. The relationship between the $k_{Q_{clin},Q}^{f_{clin},f_{ref}}$ factor and the dose homogeneity index investigated could help with determining suitable reference fields (PCSR fields) when implementing the Alfonso et al.^(1)^ formalism. This work was carried out due to the lack of investigations into collapsed versus rotated IMRT QA, particularly the lack of research into the effects, if any, of the QA method on the deviation of the dosimetry from the reference conditions (i.e., the use of small fields and comparing them to $10\times 10\;{\text{cm}}^{2}$). This would help clinics perform more accurate QA on IMRT plans, quantitatively compare results, and ultimately improve the reliability of patient treatments.

## II. MATERIALS AND METHODS

MMCTP is a radiotherapy research platform, which enables comparison and analysis of dose distributions from both treatment planning systems and quality assurance measurements, with values calculated with Monte Carlo simulations on a common independent platform.

The MMCTP system^(12)^ was used in this work to simplify and speed up the Monte Carlo simulation process. It automatically generates the files required for the Monte Carlo simulation process from the imported treatment planning files. The system also automatically submits the jobs to the remote cluster, and downloads and imports the results once the simulation is complete.

Once an accurate linear accelerator model has been commissioned, the BEAMnrc^13^, ^14^ input file can be saved by the user as a template input file on which the patient‐specific accelerator model is based in further simulations. These files are linked within MMCTP to a specific treatment machine and energy. The DOSXYZnrc part of the EGSnrc code^(15)^ is used to calculate the dose scored in a phantom. The phantom can be defined using MMCTP whether it is based on CT data or a user defined Cartesian phantom.

The EGSnrc^(16)^ egs_chamber code^(17)^ was also used, which is an ${\text{egs}}^{++}$ ^(18)^ user code designed for chamber in phantom calculations. The user code, which defines the sources and geometries, is written in C++ and is connected with the MORTRAN‐programmed EGSnrc back end which deals with the involved transport physics. The egs_chamber user code is similar to the old cavity^(18)^ user code, but it implements three new variance reduction techniques: photon cross‐section enhancement (XCSE), intermediate phase‐space storage (IPSS), and correlated sampling (CS) (which is the most powerful).

### A. Measurements

Measurements were performed using a Farmer‐type ionization chamber inserted in a tightly fitting hole in a $30\times 30\times 17\;{\text{cm}}^{3}$ Solid Water phantom (Gammex rmi, Middleton, WI), with the chamber at isocenter and perpendicular to the linac axis. Deliveries used gantry positions rotated to the planned delivery angles, as well as delivery from one angle (SGAC technique). These were of the same patient plan beams with just the gantry angle being altered using a Varian Clinac 2100 linac (Varian Medical Systems, Palo Alto, CA). The verification plan was recalculated using the beams of the patient plan. For each measurement, the phantom position was adjusted to change the chamber position as prescribed in the treatment planning system. The charge was recorded beam by beam and added after the measurements. The output stability was monitored by checking the chamber reading for a $10\times 10\;{\text{cm}}^{2}$ field before and after taking the actual measurements. The change in output was corrected. Leakage current has been measured and accounted for in the measurements, also. Stem effect is not included in the measurement, but it is assumed to be negligible for high‐energy photon beams, as investigated by Ma and Nahum.^(19)^ The cable effects were minimized by measuring the polarity effect of the chamber readings for each IMRT field in the rotated and collapsed deliveries. Dose volume statistics in the chamber volume, as well as dose distributions, were recorded from the treatment planning system.

### B. Patient treatment plans

The 20 patient plans were recalculated using the EGSnrc Monte Carlo code. The plans were for a variety of treatment sites: 15 ear, nose and throat (ENT), three head and neck, one abdominal, and one anal canal. The number of beams per plan ranged from seven up to 19 and were all for 6 MV photon beams. Clinical composite nonstandard field deliveries can generally be carried out using a photon beam having a nominal 6 MV energy,^(20)^ and so the effect of different energies has not been investigated. The treatment plans were all dynamic IMRT plans and the number of segments or beamlets per beam varied greatly. The 20 patients comprised of ten patients who were the first ten patients for whom QA was carried out when the clinic first moved to the Eclipse 8.0 TPS (Varian Medical Systems) in order to see what the difference was between collapsed and rotated QA. In Nomos Corvus 6.0 (Best nomos, Pittsburg, PA), the collapsed QA option was not available, so the collapsed option was not used until the change was made to Eclipse. The other ten patients were chosen as those for whom QA was first performed as collapsed and then repeated with rotated as they failed the collapsed QA procedure. The treatment planning system used was Eclipse using pencil beam convolution without corrections with all calculations done with homogeneous calculation.

### C. BEAMnrc and DOSXYZnrc Monte Carlo simulations

Plan data for the collapsed and rotated plans were imported into MMCTP from the DICOM files created by the treatment planning system. This patient plan data provided information on the structure, beam configuration, and calculated dose. The MMCTP system adjusts the template files using the data from the DICOM plan information to create the plan‐specific input files necessary for the Monte Carlo simulations using the EGSnrc codes. Monte Carlo simulations were submitted to a remote cluster. Previous tuning^(21)^ provided a refined BEAMnrc linear accelerator model that agreed with measured dose profile curves to an accuracy of within 2% or 3 mm for square fields with sides of 3 cm up to 30 cm and within 1% for the output factors for field sizes of $40\times 40\;{\text{cm}}^{2}$, $30\times 30\;{\text{cm}}^{2}$, $5\times 5\;{\text{cm}}^{2}$, $4\times 4\;{\text{cm}}^{2}$, and $3\times 3\;{\text{cm}}^{2}$. The initial electron energy and FWHM of the radius of the initial electron beam incident on the target was varied to find the percentage depth dose, dose profile curves, and output factors that match the hospital‐measured data, providing output factors that match within 1% of measured output factor values.

Simulations were run in two steps. Firstly, BEAMnrc models the photon production and particle transport in the accelerator and creates a phase space file at 70 cm SSD. Secondly, the DOSXYZnrc code models the particle transport and dose deposition in the phantom and the surrounding air. The simulations were run in two parts as a time‐saving measure because the same phase space files could be used as an input to the DOSXYZnrc simulations for both the rotated and collapsed simulations and later for the egs_chamber rotated and collapsed dose calculations. Directional bremsstrahlung splitting (DBS) of 1000 was used in the simulations and photon splitting of 100 was used in DOSXYZnrc to increase the calculation efficiency.^(17)^ ECUT and PCUT were set to 0.7 and 0.01 MeV, respectively.

The dose‐scoring phantom for the DOSXYZnrc simulations was generated from the CT data imported into the MMCTP program. The CT data showed a scan of a $30\times 30\times 17\;{\text{cm}}^{3}$ Solid Water phantom with the chamber centrally located within it, as used for the dose measurements. The material within the phantom was set to water and the surrounding material was set to air. The dose scoring voxels were set to a size of $0.3\times 0.3\times 0.3\;{\text{cm}}^{3}$, as this is what is used clinically.

On completion, the dose files output from DOSXYZnrc are downloaded from the remote cluster and read into MMCTP. MMCTP converts the dose‐per‐incident particle to Gy using a calibration factor obtained from a $10\times 10\;{\text{cm}}^{2}$ field run. This conversion allows for direct comparison between the Monte Carlo calculated doses and those obtained from the treatment planning system.

Once this is completed for each beam in the plan, MMCTP adds together the dose in each voxel from the different beams to obtain the total dose from the plan. DVH data and maximum, minimum, and average doses are then derived for the chamber volume allowing for a comparison with measured values. This process was repeated for the plans for each of the 20 patients in this study.

### D. EGS_chamber Monte Carlo simulations

In order to investigate the effects of not explicitly modeling the chamber, the egs_chamber code was used to recalculate the dose. The same phase space files that were previously generated with the BEAMnrc code were used as the source for these simulations. The Exradin A12 chamber (Standard Imaging Inc., Middleton, WI) was modeled explicitly at 8.5 cm depth in a $30\times 30\times 17\;{\text{cm}}^{3}$ water phantom surrounded by air (i.e., the same setup as was used for the measurements).

The Monte Carlo simulations were repeated; however, this time, it was required that the chamber was modeled in full detail with the air cavity, wall, and electrode materials, and dimensions modeled as accurately as possible to allow for an analysis of the effects of the air perturbation and pixelization of the chamber when the CT image is imported into the TPS and the previous Monte Carlo calculations. In order to do this, the ${\text{egs}}^{++}$ egs_chamber code needed to be utilized, as it is the most efficient method within the EGSnrc package of calculating the dose to the active air cavity volume of a realistic ion chamber model. While in DOSXYZnrc the chamber could have been modeled by assigning the required media to the relevant voxels in the phantom, it would be very difficult to model the chamber to any great accuracy given that the voxel sizes were $0.3\times 0.3\times 0.3\;{\text{cm}}^{3}$ and the active volume of the chamber used is only $0.65\;{\text{cm}}^{3}$.

One of the advantages of the egs_chamber code, over the ${\text{egs}}^{++}$ cavity code is the use of photon cross‐section enhancement (XCSE). XCSE is the implementation of photon splitting on a region‐by‐region basis. This means that photon splitting can be implemented in the region of the chamber and the region surrounding it, and time is not wasted simulating the tracks of extra photons and electrons that will most likely not end up contributing to the calculated dose, which is particularly the case when the dose is being calculated to a volume which is substantially smaller than the irradiated volume. In order to implement XCSE, a special XCSE region was defined surrounding the chamber region, in which photon splitting was turned on. The optimum thickness of the XCSE shell surrounding the chamber has been found to be 1 cm.^(17)^

**Figure 1:**
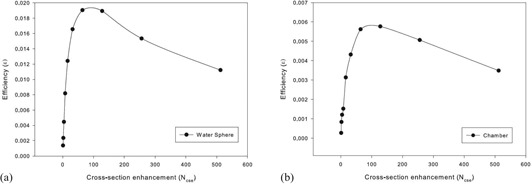
The efficiency of the egs_chamber simulations with varying $N_{\text{cse}}$ values for the photon cross‐section enhancement for (a) the water sphere and (b) the chamber simulations.

These simulations were carried out with ESAVE set to 0.1 MeV, range rejection set to 256, and $N_{\text{cse}}$ set to 128 for the chamber simulations and to 64 for the water sphere simulations, as these were found to be the optimum values for efficiency, as shown in Fig. 1. Range rejection‐based Russian roulette was set to 256, and the EGS physics parameters were kept as the default parameters.

Figure 1 depicts the results of the tests to determine the optimum enhancement factor, $N_{\text{cse}}$, for the cross‐sectional enhancement regions. The tests were run on the (a) water sphere and (b) chamber model, in a water phantom, and the range rejection‐based Russian roulette factor was set to 256 for the simulations for all enhancement factors, except for the simulation with an enhancement factor of 512 where it was set to 512, as the rejection must be larger or equal to the largest XCSE factor used. The efficiency was calculated using the equation:

(1) $$\varepsilon =\frac{1}{T\cdot \sigma^{2}}$$

where the efficiency (*ε*) is calculated using the CPU time required for the simulation in seconds (T) and the estimated percentage uncertainty in the resulting dose (σ).^(17)^

**Figure 2:**
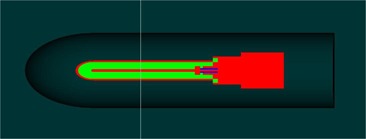
The egs_chamber ion chamber model as used for the simulations with the XCSE region visible around the chamber. The red material is C552 Shonka air‐equivalent plastic, the green is air, the dark gray is water, and the blue is Teflon.

The dose scoring region used for these simulations was the active air cavity volume of the Exradin A12 Farmer‐type ion chamber. The chamber was created in full detail using the ${\text{egs}}^{++}$ geometry definitions to produce the model, as shown in Fig. 2. The dimensions and components were modeled based on the chamber specifications obtained from the manufacturer's blueprint.

### E. $k_{Q_{clin},Q}^{f_{clin},f_{ref}}$ value

A $k_{Q_{clin},Q}^{f_{clin},f_{ref}}$ value is calculated for each plan to correct the measured values for variations in the stopping power ratio and perturbation correction factors for the small, irregular IMRT fields when compared with the reference $10\times 10\;{\text{cm}}^{2}$ field. The $k_{Q_{clin},Q}^{f_{clin},f_{ref}}$ value was calculated for each plan using the equation:

(2) $$k_{Q_{clin'Q}}^{f_{clin'}f_{ref}}=\frac{\left(D_{w,Q_{clin}}^{f_{clin}}/D_{air,Q_{clin}}^{f_{clin}}\right)}{\left(D_{w,Q_{ref}}^{f_{ref}}/D_{air,Q_{ref}}^{f_{ref}}\right)}$$

where $D_{w,Q_{clin}}^{f_{clin}}$ is the dose from the clinical IMRT photon field from each plan to a water sphere of equal volume to the active air cavity volume of the ion chamber, embedded at 8.5 cm depth in a water phantom of $30\times 30\times 17\;{\text{cm}}^{3}$ at 100 cm SAD;^(1)^ $D_{air,Q_{clin}}^{f_{clin}}$ is the dose from the clinical IMRT photon field from each plan to the air cavity volume of the ion chamber model embedded in the water phantom, again at 100 cm SAD; $D_{w,Q_{ref}}^{f_{ref}}$ is the dose from the $10\times 10\;{\text{cm}}^{2}$ reference field to the water sphere; and $D_{air,Q_{ref}}^{f_{ref}}$ is the dose from the $10\times 10\;{\text{cm}}^{2}$ reference field to the air cavity volume of the ion chamber model embedded in the water phantom at 100 cm SAD. All of these values are obtained from Monte Carlo simulations of the IMRT and $10\times 10\;{\text{cm}}^{2}$ fields using the egs_chamber code.

The $k_{Q_{clin},Q}^{f_{clin},f_{ref}}$ value can then be used to convert measurements in an IMRT clinical field using a chamber calibrated in a $10\times 10\;{\text{cm}}^{2}$ field to dose to water as follows:

(3) $$D_{w,Q_{clin}}^{f_{clin}}=M_{Q_{clin}}^{f_{clin}}\cdot N_{D,wQ_{0}}\cdot k_{Q,Q_{0}}\cdot k_{Q_{clin'}Q}^{f_{clin'}f_{ref}}$$

where $D_{w,Q_{clin}}^{f_{clin}}$ is the absorbed dose to water at a reference point in a phantom for a clinical field $f_{clin}$ of quality $Q_{clin}$ and in the absence of the chamber; $M_{Q_{clin}}^{f_{clin}}$ is the reading of the dosimeter in the field $f_{clin}$ corrected for influence quantities, such as pressure, temperature, incomplete charge collection, and polarity effects; $N_{D,w,Q_{0}}$ is the calibration coefficient in terms of absorbed dose to water for an ionization chamber at a reference beam quality $Q_{0}$ (usually Co60); $N_{D,w,Q_{0}}$ is measured at the standards laboratory for a reference field of size $10\times 10\;{\text{cm}}^{2}$; $k_{Q,Q_{0}}$ is the beam quality correction factor, which corrects for the differences between the reference beam quality $Q_{0}$ at the standards laboratory and the beam quality *Q* of the conventional field $f_{ref}$ and is the correction factor calculated in this work to account for the difference between the responses of the ionization chamber in the reference $10\times 10\;{\text{cm}}^{2}$ field $f_{ref}$ and the clinical IMRT field $f_{clin}$.

### F. Plan complexity metrics

A number of different complexity metrics were utilized to evaluate and quantify the complexity of the IMRT plans used in this study and, hence, examine the effect of plan complexity on dose measurement and calculation.

#### F.1 Dose homogeneity index (HI)

The homogeneity index (HI) is a measure of the dose homogeneity within the chamber volume, and is calculated as defined by Wu et al.^(22)^ as:

(4) $$\text{Homogeneity Index}\;(\text{HI})=\frac{D_{2\%}-D_{98\%}}{D_{\text{average}}}$$

where $D_{2\%}$ is the dose to 2% of the chamber volume as displayed on the cumulative DVH, $D_{98\%}$ is the dose to 98% of the chamber volume as displayed on the cumulative DVH, and $D_{\text{average}}$ is the average dose to the chamber volume. $D_{2\%}$ and $D_{98\%}$ are the near maximum and near minimum doses, respectively, of the chamber volume. These concepts are defined in ICRU Report 83.^(23)^ The greater the HI value, the more heterogeneous the dose within the chamber volume.

#### F.2 Dose conformity index (COIN)

There are many differing definitions of the conformity index of a dose distribution as discussed by Feuvret et al.^(24)^ The one that has been used in this work is a slightly modified version of one of these and is defined as:

(5) $$\text{Conformity Index}(\text{COIN})=\frac{{\text{PTV}}_{\text{small},\text{RI}}}{{\text{PTV}}_{\text{small}}}\times \frac{{\text{PTV}}_{\text{small},\text{RI}}}{{\text{PTV}}_{\text{COIN},\text{RI}}}$$

where ${\text{PTV}}_{\text{small}}$ is the volume of the chamber contour at the center of the phantom, *RI* is the reference isodose which has been defined here as the average dose to the ${\text{PTV}}_{\text{small}}$ volume, ${\text{PTV}}_{\text{small},\text{RI}}$ is the volume of the small target which is covered by the reference isodose, and ${\text{PTV}}_{\text{COIN},\text{RI}}$ is the volume of the added structure of 1.5 cm border surrounding the chamber volume, which is covered by the reference isodose. When using the conformity index to evaluate clinical treatment plans, a larger value for the conformity index would be considered better (an ideal value of 1 would indicate precise coverage of the target volume with absolutely no dose to the surrounding tissues). However, here a larger value (closer to 1) is considered worse, as it means that there is greater dependence on accurate chamber positioning (as there would be a large dose gradient surrounding the chamber). A low value would imply that a larger portion of the region surrounding the chamber is also covered by the reference isodose and, hence, chamber positioning is less crucial.

#### F.3 Plan modulation complexity score (MCS)

The modulation complexity score (MCS),^(25)^ for a plan with *J* number of beams, is calculated by:

(6) $$MCS_{plan}=\sum_{j=1}^{J}MCS_{bea\mathrm{m j}}\times \frac{MU_{bea\mathrm{m j}}}{MU_{plan}}$$

where

(7) $$MCS_{beam}=\sum_{i=1}^{I}AAV_{segmen\mathrm{t i}}\times LSV_{segmen\mathrm{t i}}\times \frac{MU_{segmen\mathrm{t i}}}{MU_{beam}}$$

for *I* segments per beam. The weight of each beam is taken into account by weighting each of the scores based on the number of monitor units delivered by each beam.

The aperture area variability (AAV), with A number of leaves in the leaf bank, can be calculated for each segment of the beam as follows:

(8) $$AAV_{segment}=\frac{\sum_{a=1}^{A}<pos_{a}{>}_{lef\mathrm{t b}an\mathrm{k p}lan}-<pos_{a}{>}_{righ\mathrm{t b}an\mathrm{k p}lan}}{{\sum_{a=1}^{A}<\text{max}(pos_{a}){>}_{lef\mathrm{t b}an\mathrm{k b}eam}-<\text{max}(pos_{a})>}_{righ\mathrm{t b}an\mathrm{k b}eam}}$$

The leaf sequence variability (LSV), with N number of open leaves in the leaf bank, can be calculated for each segment of the beam as follows:

(9) $$\begin{array}{ccc} LSV_{segment} & = & {〈\frac{\sum_{n=1}^{N}\left(pos_{\max}-(pos_{n}-pos_{n+1})\right)}{N\times pos_{\max}}〉}_{lef\mathrm{t b}ank}\times \\ & & {〈\frac{\sum_{n=1}^{N}\left(pos_{\max}-(pos_{n}-pos_{n+1})\right)}{N\times pos_{\max}}〉}_{rightbank} \end{array}$$

where

(10) $$pos_{\max}={〈\text{max}(pos_{N\varepsilon n})-\text{min}(pos_{N})〉}_{\text{leaf bank segment}}$$

This is calculated using the coordinates of the leaf positions (pos).

## III. RESULTS & DISCUSSION

### A. DoSXYZnrc simulations

**Figure 3:**
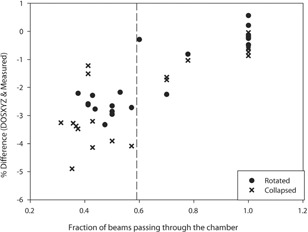
How the fraction of beams passing through the chamber affects the difference between the Monte Carlo calculated dose and the measured dose.

There is good agreement between Monte Carlo dose‐to‐water calculations and measured doses once the fraction of beams passing through the chamber is >0.6 (i.e., when more than 60% of the beams in a given plan pass directly through the ionization chamber as opposed to not intersecting the chamber and just passing through a part of the phantom). The number of beams intersecting the chamber was determined upon visual inspection of the dose distribution on the treatment planning system and a beam was considered to intersect the chamber if its dose distribution intersected the chamber contour. When this occurs, the DOSXYZnrc‐calculated doses for the collapsed plans agree with the corrected measured values to within 1.73%, and the DOSXYZnrc‐calculated doses for the rotated plans agree with corrected measurements to within 2.5% (Fig. 3).

There are several possible reasons for the greater differences when the number of beams tangential to the chamber volume is >40%. Calculations in these beams are very sensitive to the accuracy of the beam model. Secondly, these are dose‐to‐water calculations and we are not modeling the chamber in full detail; this may influence the accuracy of the results, specifically in these beams. The egs_chamber code was used to model the chamber in full detail to investigate these effects further.

**Figure 4:**
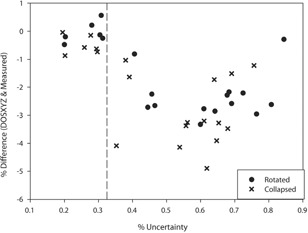
Effect of the uncertainty in the dose in the chamber, as calculated with Monte Carlo, on the difference between the Monte Carlo‐calculated dose and the measured dose.

It is believed that the greater difference between Monte Carlo calculated doses and measured doses for the plans where fewer beams intersect the chamber could also be due to the fact that these plans have greater uncertainty in the dose in the chamber. It can be seen in Fig. 4 that the greater uncertainty leads to greater differences.

When the uncertainty in the Monte Carlo dose is approximately <0.3%, then the collapsed MC doses agree with the measured doses within 1% and the rotated MC doses agree within 0.5%. The better agreement for the rotated doses is expected to be due to the fact that the uniformity of the dose in the chamber for the rotated plans is generally better. The uncertainty value of <0.3% only occurs when 100% of the beams in the plan pass through the chamber (Fig. 4). The uncertainty is calculated as:

(11) $$Tota\mathrm{l u}ncerta\text{int}y=\sqrt{\frac{\sum_{i=1}^{n}i^{2}}{n}}$$

where *i* is the uncertainty of the dose within the chamber contour from each beam in the plan and *n* is the total number of beams in the plan.

**Figure 5:**
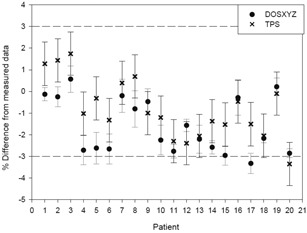
Difference between the Monte Carlo‐calculated dose and the measured dose on a patient‐by‐patient basis for the rotated plans.

**Figure 6:**
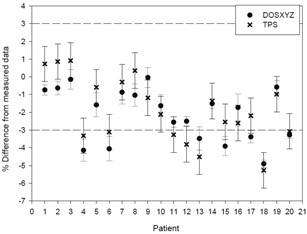
Difference between the Monte Carlo‐calculated dose and the measured dose on a patient‐by‐patient basis for the collapsed plans.

From Figs. 5, 6, and in particular 7, it can be seen that the Monte Carlo‐calculated doses provide better agreement to the measured dose values than the TPS in 66% of cases or 83% of rotated cases when the Type A uncertainty in the Monte Carlo DOSXYZ value is less than 0.4%. The difference values are mostly negative as the $k_{Q_{clin},Q}^{f_{clin},f_{ref}}$ values are mostly less than 1 for the range of plans investigated in this work, especially for the collapsed plans investigated.

From Fig. 6, it can be seen from the first ten patients, only patients 4 and 6 have poor agreement between measurements versus Monte Carlo calculations and measurements versus TPS calculations (>3% difference). When the complexity of the plans was investigated, only patient 6 had a low COIN value (<0.05) and patient 4 had a larger HI value (>0.15 for collapsed plans). Patient 10 had a larger HI value (0.16) also, but not as high as patient 4 (0.21). All the first ten patients agree within 3% for the rotated values (Fig. 5).

From the second ten patients (the ones who failed collapsed QA), five have >3% difference between measurements and TPS (i.e., failed the absolute dose measurement). These are patients 11, 12, 13, 18, and 20. Of these, patients 12 and 20 had larger HI values and patients 13 and 18 had low COIN values. Though it is not clear why patient 11 failed, it could just be an inaccurate measurement, as this analysis was carried out retrospectively.

Based on the Monte Carlo and measured value differences, patients 15 and 17 would also have failed and also have larger HI values. For the rotated measurements, the TPS values all agree within 3%, except for patient 20 whose plan has a low COIN value in the rotated setup. Monte Carlo calculations all agree with measurements to within 3% except patient 17, whose plan has a slightly larger HI value but not the highest; it does, however, have the lowest MCS score — making it the most complex plan.

### B. Phase 2 — egs chamber simulations

The measured values corrected with the $k_{Q_{clin},Q}^{f_{clin},f_{ref}}$ factors lead to improved agreement (compared to the agreement between egs_chamber and uncorrected measurements) between the measured values and egs_chamber dose‐to‐water values in the majority of cases — 14 out of 20 for collapsed delivery and 11 out of 20 for rotated beam delivery (Fig. 8). Due to the nature of clinical measurements, there will always be differences between the measured and Monte Carlo simulated values; so, if the correction factor is small, it is reasonable that it would make the agreement between the measurements and simulated values worse in some cases. This is one possible reason for the reduced effect in the rotated beam delivery, as there was already good agreement in these cases and the fact that the $k_{Q_{clin},Q}^{f_{clin},f_{ref}}$ values are closer to 1 in these cases, so have less of an effect. The main observation is that the percent relative root mean square difference is better when the correction is implemented. This holds for both rotated and collapsed delivery with values for the collapsed delivery of 3.9 and 3.7 for the uncorrected and corrected measurements, respectively, and values for the rotated delivery of 3.0 and 2.7 for the uncorrected and corrected measurements, respectively, for the percent relative root mean square difference.

**Figure 7:**
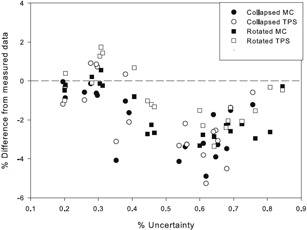
Effects of the Monte Carlo Type A dose calculation uncertainty from DOSXYZnrc on the agreement of the dose calculation methods.

**Figure 8:**
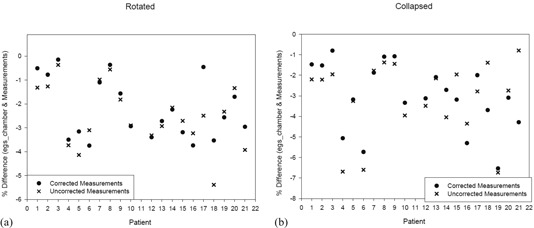
The percent difference in dose values obtained from the egs_chamber simulations and the corrected with $k_{Q_{clin},Q}^{f_{clin},f_{ref}}$ and uncorrected measurement values for both (a) the rotated and (b) the collapsed beam delivery methods.

**Figure 9:**
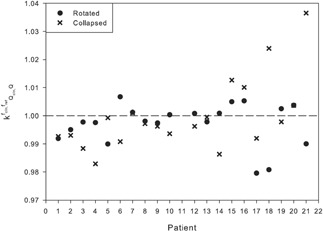
The $k_{Q_{clin},Q}^{f_{clin},f_{ref}}$ values for each of the 20 patients for both the collapsed and rotated beam deliveries.

With values for $k_{Q_{clin},Q}^{f_{clin},f_{ref}}$ deviating from the ideal value of 1 by as much as 3.6% for collapsed delivery but a maximum deviation of 2% for rotated delivery (Fig. 9) would imply that IMRT QA using rotated delivery is a better option, as the measured dose should be a closer match to delivered dose based on the results of this cohort of patients.

From Table 1 it can be seen that the average difference from measured values is always greater for the collapsed technique than for the rotated technique. The $k_{Q_{clin},Q}^{f_{clin},f_{ref}}$ correction factor improves the agreement between measured values and Monte Carlo calculations. The egs_chamber values have worse agreement, but this could be due to the inaccuracies in the DOSXYZ model (due to pixelization of the chamber volume) concealing inaccuracies in the measured dose values. It is, however, still necessary to perform the more detailed simulation, as it is the only way of accurately calculating the $k_{Q_{clin},Q}^{f_{clin},f_{ref}}$ factors used to correct the measurements for comparison with DOSXYZnrc dose values. The greater difference for the collapsed measurements could also be due to the inherent experimental uncertainties that could contribute differently in the rotated versus the collapsed method. For example, a small source‐to‐surface distance error would partially cancel out in the rotated delivery method, whereas in the collapsed method, it would have a systematic contribution to the error.

**Table 1:** A summary of the average differences between Monte Carlo and measured dose values

	*Rotated Average (Std Dev)*	*Collapsed Average (Std Dev)*
DOSXYZ	−1.602 (1.272)	−2.134 (1.492)
DOSXYZ (corrected)	−1.311 (1.510)	−2.085 (2.075)
egs_chamber	−2.499 (1.300)	−3.039 (1.819)

### C. Effects of plan complexity

**Figure 10:**
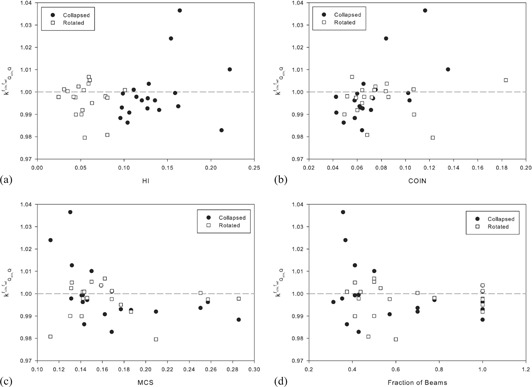
The effect of the different plan complexity metrics investigated on the $k_{Q_{clin},Q}^{f_{clin},f_{ref}}$ values. The complexity metrics investigated were: homogeneity index (a), conformity index (b), modulation complexity score (c), and the fraction of beams that directly intersect the chamber (d).

From Fig. 10, the effects of the different plan complexity metrics investigated can be seen on the $k_{Q_{clin},Q}^{f_{clin},f_{ref}}$ values. In Fig. 10(a), it can be seen that the more heterogeneous the dose within the chamber, the more the $k_{Q_{clin},Q}^{f_{clin},f_{ref}}$ values deviate from the ideal value of 1. If HI is less than 0.05, then the $k_{Q_{clin},Q}^{f_{clin},f_{ref}}$ values do not deviate from unity by more than 1%. This is consistent with Chung et al.^(26)^ A value for HI of less than 0.05 can only be achieved with rotated delivery for the plans investigated. The $k_{Q_{clin},Q}^{f_{clin},f_{ref}}$ values for the rotated plans start to deviate more from 1 at lower HI values than for collapsed plans, but, overall, the HI values for the rotated plans are lower than those for the collapsed plans and the $k_{Q_{clin},Q}^{f_{clin},f_{ref}}$ values for the rotated plans as a whole deviate less from 1.

In Fig. 10(b) it can be seen that the $k_{Q_{clin},Q}^{f_{clin},f_{ref}}$ values deviate more from 1 for larger COIN values. This is again in agreement with the results obtained by Chung and colleagues. This effect is more pronounced in the collapsed plans, which is most likely due to the more heterogeneous dose distributions in this type of beam setup.

In Fig. 10(c) it can be seen that the $k_{Q_{clin},Q}^{f_{clin},f_{ref}}$ values deviate more from 1 for more complex plans (i.e., a lower MCS value). This is to be expected as more complex plans are classed as ones where the beamlets are more complex in shape (i.e., the least like the square reference field). This is also much more pronounced for the collapsed beams, but this is again presumably due to the higher dose inhomogeneity within the chamber in this delivery format.

In Fig. 10(d) it can be seen that the $k_{Q_{clin},Q}^{f_{clin},f_{ref}}$ values deviate more from 1 when the fraction of beams that directly intersect the chamber volume is lower. This is in agreement with what has been previously reported by Capote et al.^(27)^ where they noted the largest correction values when the chamber is located outside the beamlet.

**Figure 11:**
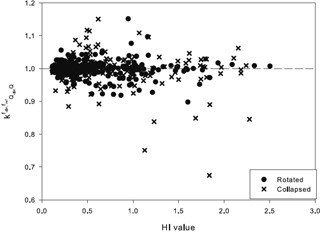
The $k_{Q_{clin},Q}^{f_{clin},f_{ref}}$ values for each of the 245 beams for both the collapsed and rotated beam deliveries from all 20 patients.

Each of the 245 beams from the 20 patients was further investigated individually. From this beam‐by‐beam analysis, the $k_{Q_{clin},Q}^{f_{clin},f_{ref}}$ values show a much greater spread in the values, particularly at the higher values for the dose homogeneity index (Fig. 11). This is due to the fact that, for individual beams, there are more obvious deviations from CPE, which is reflected in the $k_{Q_{clin},Q}^{f_{clin},f_{ref}}$ values deviating more from 1.

From these results it can be seen that more complex plans (i.e., plans with lower MCS values, fewer beams intersecting the chamber, and less homogeneous dose within the chamber) are more likely to have poor agreement between measured data and the TPS data and are more likely to need an added correction factor $k_{Q_{clin},Q}^{f_{clin},f_{ref}}$ when converting the measured charge to absolute dose. If HI is small, then the IMRT QA is easier and the difference between the measurement and calculation is smaller. This would suggest that a region of low HI (preferably less than 0.05) should be chosen to place the chamber when doing QA measurements.

## V. CONCLUSIONS

This work shows the potential of using Monte Carlo techniques to assess and validate dosimetric quality assurance techniques, which is of particular importance when assessing current QA techniques to decide on a method of best practice or when implementing new treatment modalities. Based on this investigation, it is apparent that rotated beam delivery for absolute dose measurement is more consistent, as the $k_{Q_{clin},Q}^{f_{clin},f_{ref}}$ values deviate less from 1 than collapsed mode. However, if a collapsed technique must be used, it would be worthwhile to take into account the homogeneity of the dose in the region in which the measurement is to be performed, the complexity of the plan, and the resulting dose distribution.

## ACKNOWLEDGMENTS

We acknowledge the help of Brian Hooten at Standard Imaging for providing blueprints for the ionization chamber model.
